# Pancreatic schwannoma: A case report and review of the literature

**DOI:** 10.3892/ol.2014.2578

**Published:** 2014-09-30

**Authors:** NAZAN CILEDAG, KEMAL ARDA, MUSTAFA AKSOY

**Affiliations:** 1 Department of Radiology, Ankara Oncology Research and Education Hospital, Demetevler, Ankara 06200, Turkey; 2 Department of Radiology, Atatürk Research and Education Hospital, Eskişehir Yolu, Ankara 06800, Turkey; 3 Department of Anesthesia, Atatürk Research and Education Hospital, Eskişehir Yolu, Ankara 06800, Turkey

**Keywords:** schwannoma, magnetic resonance imaging, cystic mass, neurilemmoma, pancreas

## Abstract

Schwannoma or neurilemmoma is a well-defined, benign tumor, which arises from neural crest cells and surrounds the nerve sheath. It is rare neoplasm that is typically found in the extremities, such as the thorax, head, neck, pelvis and rectum. Schwannoma localized to the pancreas is particularly rare and only a limited number of cases have been reported in the literature to date. The present study reports the case of a 30-year-old male with pancreatic schwannoma presenting with weight loss and abdominal pain. Pancreatic schwannoma was diagnosed using magnetic resonance imaging and ultrasonography-guided biopsy, which was followed by a duodenopancreatectomy. Although pancreatic schwannomas are rare, they must be considered during the differential diagnoses of cystic pancreatic masses.

## Introduction

Schwannomas or neurilemmomas are rare neoplasms that typically occur in the peripheral nerve sheath of the extremities. However, visceral localization of these tumors, specifically pancreatic schwannomas that arise from either sympathetic or parasympathetic fibers of the pancreas, is particularly rare (1). Pancreatic schwannomas affect adults with an equal gender distribution. In the majority of cases, these tumors are well-defined, encapsulated solid masses with hemorrhage or cystic degeneration, calcification, hyalinization and xanthomatous infiltration (1–3). Imaging findings of pancreatic schwannomas with cystic degeneration may present a cystic pancreatic lesion. The present study reports a patient with a pancreatic head tumor presenting with weight loss and abdominal pain. The pancreatic head tumor was diagnosed as a schwannoma, which was considered to be a rare case with an unusual localization. The patient provided written informed consent.

## Case report

A 30-year-old male was admitted to the Ankara Oncology Research and Education Hospital (Ankara, Turkey) presenting with weight loss and abdominal pain. The patient exhibited no other systemic symptoms. On physical examination, a tender mass in the epigastrium was palpated. The laboratory examination results, including hemoglobin, liver function tests, amylase and tumor marker levels (carbohydrate antigen 19-9 and carcinoembryonic antigen) were in the normal ranges.

**Figure 1:**
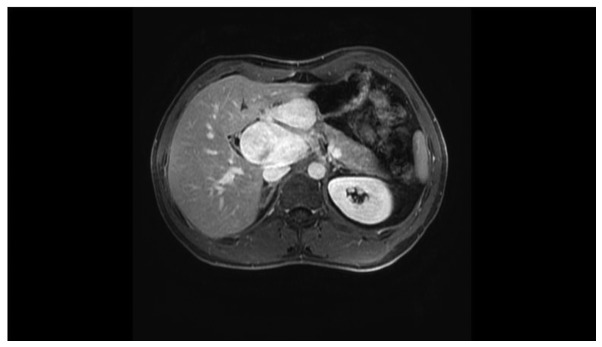
Axial T2-weighted fat saturation sequences show a marked hyperintense pancreatic head mass that abuts the hepatic artery.

**Figure 2:**
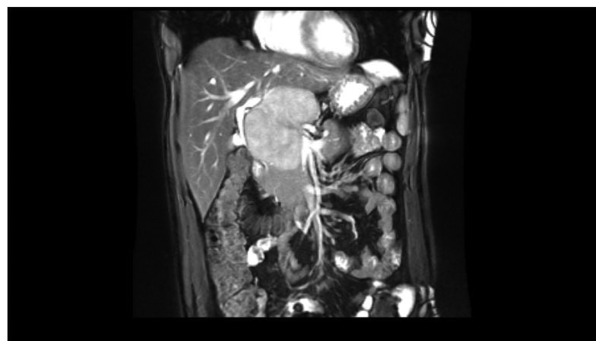
Coronal T2-weighted fat-suppressed magnetic resonance image shows a marked hyperintense pancreatic head mass.

**Figure 3:**
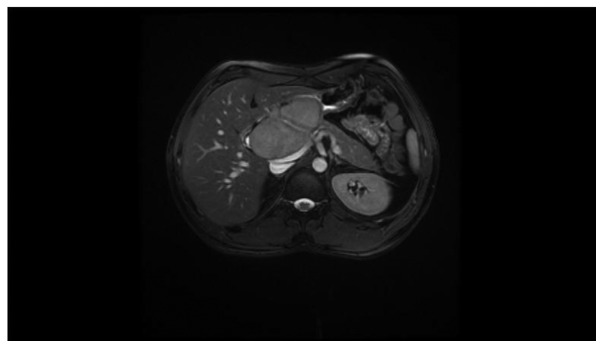
Axial gadolinium-enhanced T1-weighted fat-suppressed magnetic resonance image shows significant enhancement of the mass at the pancreas head and uncinate process.

**Figure 4:**
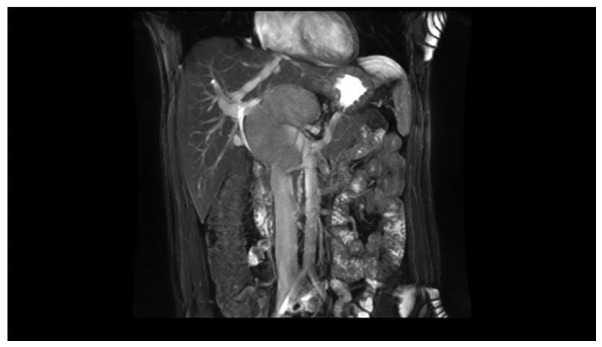
Coronal gadolinium-enhanced T1-weighted fat-suppressed magnetic resonance image shows significant enhancement of the mass at the pancreas head and uncinate process.

Abdominal ultrasonography revealed a hypoechoic mass measuring 7.6×3 cm in the pancreatic head. Upper abdominal computed tomography (CT) showed a hypodense mass measuring 10×7 cm arising from the head of the pancreas. Upper abdominal T1-weighted dynamic magnetic resonance imaging (MRI) revealed a hypointense, bilobular, contoured, encapsulated mass measuring 8.7×9 cm, which exhibited cystic components arising from the head and the uncinate process of the pancreas and portal hilus; the mass encased the superior mesenteric artery and laterally replaced the portal vein. Following the administration of gadolinium, an early and persistent enhanced signal was noted in the T2-weighted fat saturation sequences (Figs. 1 and 2), and the lesion was markedly hyperintense (Figs. 3 and 4). Based on the patient’s history, and the clinical and imaging findings, an ultrasonography-guided Tru-cut needle (WestCott 16G, Beckton Dickinson, Downers Grove, IL, USA) biopsy was performed and pathological evaluation showed characteristic spindle cells and strong positive immunoperoxidase staining for S-100 protein, which was consistent with schwannoma. Therefore, a duodenopancreatectomy was performed.

## Discussion

Schwannoma or neurilemmoma are rare, well-defined, benign encapsulated, slow growing tumors arising from Schwann cells that encase the peripheral nerves (1–3). Extracranial schwannomas typically occur in the extremities, however, are also found in the trunk, head and neck, pelvis and rectum (4–10). Intra-abdominal, retroperitoneal and particularly intra-pancreatic presentation of schwannoma is extremely rare (9,10). The number of cases of schwannoma located in the small bowels, bile ducts, pelvis and sacrum are currently limited (6,11) with <26 cases of pancreatic schwannoma reported in the literature to date. These tumors vary considerably in size, ranging from 1.5 to 20.0 cm in diameter and the majority of the tumors are located in the head (38%) and body (25%) of the pancreas. Half of the reported schwannomas are cystic and 5% of schwannomas are associated with neurofibromatosis type 1.

Typical CT findings of pancreatic schwannomas are similar to non-pancreatic schwannomas and demonstrate a well-defined, encapsulated, hypointense solid mass with hemorrhage or cystic degeneration, calcification or hyalinization (1–3,12). Cystic formation may mimic cystic pancreatic lesions, such as neuroendocrine tumors, cystadenoma, cystadenocarcinoma, intraductal papillary mucinous tumor, lymphangiomas and pancreatic pseudocysts (13).

Characteristic MRI findings of these tumors include typical encapsulation, hypointensity on T1-weighted images and hyperintensity on T2-weighted images (13). MRI may also differentiate pancreatic schwannoma from adenocarcinoma due to the characteristic hyperintensity on T2-weighted images and marked enhancement of the lesion in comparison with the remainder of the pancreas.

In the present case, the tumor was an encapsulated pancreatic mass with cystic components. Although CT and MRI may aid in the differential diagnosis, a definitive diagnosis of pancreatic schwannoma requires histopathological examination. Microscopically, schwannomas are strongly positive for S-100 protein, vimentin and cluster of differentiation 56, however, are negative for other tumor markers (14). Surgical excision with a close follow-up and surveillance remain the mainstay treatment method for pancreatic schwannomas.

In conclusion, the diagnosis of pancreatic schwannomas, although they are rare, must be considered in the differential diagnosis of well-defined, encapsulated cystic lesions of the pancreas.

## References

[1] Tofigh AM, Hashemi M, Honar BN, Solhjoo F (2008). Rare presentation of pancreatic schwannoma: a case report. J Med Case Rep.

[2] Di Benedetto F, Spaggiari M, De Ruvo N (2007). Pancreatic schwannoma of the body involving the splenic vein: case report and review of the literature. Eur J Surg Oncol.

[3] Okuma T, Hirota M, Nitta H (2008). Pancreatic schwannoma: report of a case. Surg Today.

[4] Jayaraj SM, Levine T, Frosh AC, Almeyda JS (1997). Ancient schwannoma masquerading as parotid pleomorphic adenoma. J Laryngol Otol.

[5] Dayan D, Buchner A, Hirschberg A (1989). Ancient neurilemmoma (Schwannoma) of the oral cavity. J Craniomaxillafac Surg.

[6] Hide IG, Baudouin CJ, Murray SA, Malcolm AJ (2000). Giant ancient schwannoma of the pelvis. Skeletal Radiol.

[7] Graviet S, Sinclair G, Kajani N (1995). Ancient schwannoma of the foot. J Foot Ankle Surg.

[8] McCluggage WG, Bharucha H (1995). Primary pulmonary tumours of nerve sheath origin. Histopathology.

[9] Loke TH, Yuen NW, Lo KK, Lo J, Chan JC (1998). Retroperitoneal ancient schwannoma: review of clinico-radiological features. Australas Radiol.

[10] Giglio M, Giasotto V, Medica M, Germinale F, Durand F, Queirolo G, Carmignani G (2002). Retroperitoneal ancient schwannoma: case report and analysis of clinico-radiological findings. Ann Urol (Paris).

[11] Toh LM, Wong SK (2006). A case of cystic schwannoma of the lesser sac. Ann Acad Med Singapore.

[12] Tortorelli AP, Rosa F, Papa V (2007). Retroperitoneal schwannomas: diagnostic and therapeutic implications. Tumori.

[13] Ferrozzi F, Bova D, Garlaschi G (1995). Pancreatic schwannoma: report of three cases. Clin Radiol.

[14] Tan G, Vitellas K, Morrison C, Frankel WL (2003). Cystic schwannoma of the pancreas. Ann Diagn Pathol.

